# Neural-Network-Based Diagnosis Using 3-Dimensional Myocardial Architecture and Deformation: Demonstration for the Differentiation of Hypertrophic Cardiomyopathy

**DOI:** 10.3389/fcvm.2020.584727

**Published:** 2020-11-11

**Authors:** Alessandro Satriano, Yarmaghan Afzal, Muhammad Sarim Afzal, Ali Fatehi Hassanabad, Cody Wu, Steven Dykstra, Jacqueline Flewitt, Patricia Feuchter, Rosa Sandonato, Bobak Heydari, Naeem Merchant, Andrew G. Howarth, Carmen P. Lydell, Aneal Khan, Nowell M. Fine, Russell Greiner, James A. White

**Affiliations:** ^1^Stephenson Cardiac Imaging Center, Calgary, AB, Canada; ^2^Division of Cardiology, School of Medicine, University of Calgary, Calgary, AB, Canada; ^3^Libin Cardiovascular Institute of Alberta, Calgary, AB, Canada; ^4^Department of Diagnostic Imaging, University of Calgary, Calgary, AB, Canada; ^5^Department of Medical Genetics, University of Calgary, Calgary, AB, Canada; ^6^Department of Computing Science, University of Alberta, Edmonton, AB, Canada; ^7^Alberta Machine Learning Institute, Edmonton, AB, Canada

**Keywords:** machine learning, neural network, strain analysis, magnetic resonance, cardiomyopathy, hypertrophic

## Abstract

The diagnosis of cardiomyopathy states may benefit from machine-learning (ML) based approaches, particularly to distinguish those states with similar phenotypic characteristics. Three-dimensional myocardial deformation analysis (3D-MDA) has been validated to provide standardized descriptors of myocardial architecture and deformation, and may therefore offer appropriate features for the training of ML-based diagnostic tools. We aimed to assess the feasibility of automated disease diagnosis using a neural network trained using 3D-MDA to discriminate hypertrophic cardiomyopathy (HCM) from its mimic states: cardiac amyloidosis (CA), Anderson–Fabry disease (AFD), and hypertensive cardiomyopathy (HTNcm). 3D-MDA data from 163 patients (mean age 53.1 ± 14.8 years; 68 females) with left ventricular hypertrophy (LVH) of known etiology was provided. Source imaging data was from cardiac magnetic resonance (CMR). Clinical diagnoses were as follows: 85 HCM, 30 HTNcm, 30 AFD, and 18 CA. A fully-connected-layer feed-forward neural was trained to distinguish HCM vs. other mimic states. Diagnostic performance was compared to threshold-based assessments of volumetric and strain-based CMR markers, in addition to baseline clinical patient characteristics. Threshold-based measures provided modest performance, the greatest area under the curve (AUC) being 0.70. Global strain parameters exhibited reduced performance, with AUC under 0.64. A neural network trained exclusively from 3D-MDA data achieved an AUC of 0.94 (sensitivity 0.92, specificity 0.90) when performing the same task. This study demonstrates that ML-based diagnosis of cardiomyopathy states performed exclusively from 3D-MDA is feasible and can distinguish HCM from mimic disease states. These findings suggest strong potential for computer-assisted diagnosis in clinical practice.

## Introduction

The application of machine-learning (ML) techniques for computer-assisted diagnosis is an anticipated milestone for cardiac imaging. However, robust approaches to the classification of cardiomyopathy states requires concurrent consideration of architectural features as well as regional patterns of deformation. This set of requirements poses challenges for the development of ML-assisted diagnosis in cardiovascular diagnostics.

Three-dimensional myocardial deformation analysis (3D-MDA) is a recently validated (1–9) image post-processing technique for the transformation of dynamic imaging data into a standardized, time-resolved 3D model of cardiac phenotype. Using foundational techniques transferrable across cardiac magnetic resonance (CMR) (1, 4, 6–8), computed tomography (CT) (10) and 3D echocardiography (3), this offers a common approach to convert source images into data required to describe chamber phenotypes using spatially-resolved measures of wall thickness and principal strain (PS), the latter an objective measure of tissue deformation in the direction resulting from locally-engaged myocardial fibers (1, 9). We hypothesized that a ML-based model could use this standardized phenotypic data to classify cardiomyopathy state. As a sentinel demonstration of this novel paradigm, we chose to assess diagnostic accuracy of a neural network trained exclusively from 3D-MDA data (wall architecture and deformation) to distinguish hypertrophic cardiomyopathy (HCM) from its commonly encountered mimic states: cardiac amyloidosis (CA), Anderson–Fabry disease (AFD), and hypertensive cardiomyopathy (HTNcm).

## Materials and Methods

### Study Population

Patients were identified from the Cardiovascular Imaging Registry of Canada (CIROC), a prospective initiative of the Libin Cardiovascular Institute at the University of Calgary (Calgary, Alberta) (NCT04367220). Data management is executed by commercial software (cardioDI™, Cohesic Inc., Calgary). This data repository was interrogated to identify patients with a confirmed etiology of left ventricular hypertrophy (LVH), defined as an indexed LV mass index ≥2 SD above age and sex-based reference values (11) and a maximal LV wall thickness ≥13 mm by linear measurement on at least 1 short-axis (SAX) cine view. Etiology of LVH was established by review of medical records and all available diagnostic testing. HCM diagnosis required either genotype confirmation of a pathologic sarcomere protein mutation, a 1st degree relative with the same, or a maximal wall thickness ≥15 mm, plus typical mid-wall patchy fibrosis by late gadolinium enhancement (LGE) imaging and no other identifiable cause. CA diagnosis required a typical diffuse pattern of LGE with confirmatory testing by either fat pad biopsy (for light chain amyloidosis) or 99m-technetium-pyrophosphate scintigraphy imaging (for transthyretin variant amyloidosis). AFD diagnosis required positive genotype for a pathologic mutation of the alpha galactosidase encoding gene. Finally, HTNcm diagnosis required a history of hypertension for ≥10 years, no family history of HCM or AFD, and no LGE-based features suggestive of an alternate myocardial disease state.

All CMR studies were de-identified and transferred to a study server for blinded analysis. The study design was approved by the Conjoint Health Research Ethics Board at the University of Calgary and all subjects provided written informed consent. All research activities were performed in accordance with the Declaration of Helsinki.

### MRI Image Acquisition

All CMR studies were performed using 3T scanners (Prisma or Skyra, Siemens Healthineers, Erlangen, Germany). A standardized imaging protocol was performed in all patients inclusive of both cine and LGE imaging. Cine imaging was performed in sequential SAX and standard long-axis (LAX) imaging planes at end-expiration using a steady-state free precession pulse sequence. Typical imaging parameters were: repetition time 3.1 ms, 6 lines per segment, echo time 1.3 ms, flip angle 45°, field of view 276 × 360 mm^2^, matrix 156 × 192, slice thickness 6 mm, gap 2 mm, parallel imaging factor of 2, reconstructed to 30 cardiac phases. LGE imaging was performed 7-10 min following intravenous administration of 0.1 mmol/kg gadolinium chelate (Gadovist, Bayer Inc., Canada) in matched slice orientations using a standard inversion-recovery gradient pulse sequence with phase sensitive image reconstruction (12).

### Conventional Volumetric Chamber Analysis

All analyses were performed by trained core laboratory personnel blinded to clinical data. Each type of image analysis was performed independently and in random order. LV volumes and mass were calculated using commercially available software (cvi^42^, Circle Cardiovascular Inc., Calgary, Canada) from sequential SAX cine images using semi-automated tracing of the endocardial and epicardial contours to derive LV end-diastolic volume (LVEDV), LV end-systolic volume (LVESV), LV ejection fraction (LVEF), and mass. Papillary muscles were considered part of the LV mass. Chamber volumes and mass were indexed to body surface area (BSA) according to the Mostellar formula.

### 3D Myocardial Deformation Analysis

**Figure 1:**
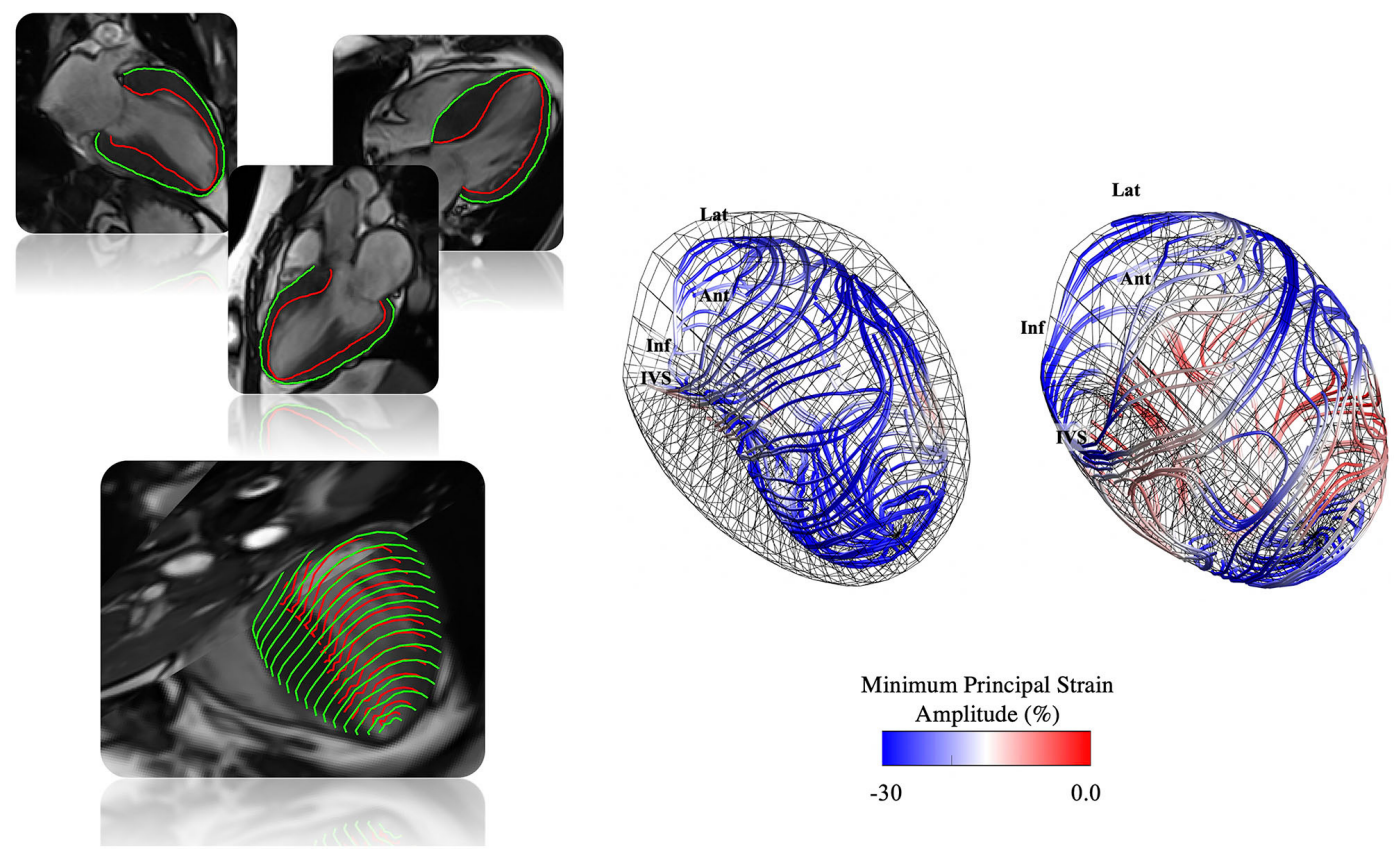
Illustration of 3D-MDA workflow. **Top left:** Endocardial and epicardial contour tracing performed at end-diastole on long-axis cine images with automated registration to short-axis images and 3D mesh generation. **Bottom left:** A 4D displacement field is generated (1, 4, 6–8, 15), and used to deform the end-diastolic phase 3D LV mesh (1, 3, 4, 6) throughout the cardiac cycle (1, 4, 6). **Right:** From this dynamic mesh model strain, conventional and principal strain quantification is performed (16, 17). In the example, principal strain amplitude and direction lines are shown for a patient with confirmed hypertrophic cardiomyopathy (HCM).

3D-MDA of the LV was performed using validated in-house software, leveraging previously established technologies for image registration, temporal tracking, mesh generation, and computation of deformation (1–4, 6, 13, 14). Briefly, a 4D displacement field is generated using optical-flow-based feature tracking (7, 8) of all pixels from co-registered multi-planar 2D cines (1, 4, 6, 7), automatically adjusting for breath-hold motion (15). This field is used to deform an end-diastolic 3D mesh model of the LV (1, 3, 4, 6), defined by ~500 nodal elements present on each of the endocardial and epicardial layers. This results in a cross-subject (and cross-modality) anatomically coherent model throughout 30 phases of the cardiac cycle (Figure 1) (1, 4, 6). Using this standardized dynamic mesh, peak-systolic strain amplitude, time to peak-systolic strain, maximum systolic and diastolic strain rates, and end-diastolic thickness are estimated from pre-defined hexahedral structured mesh elements (16, 17). For this study, corresponding analyses were expressed as (i) global mean values and (ii) segmental mean values according to the American Heart Association (AHA) standard (18). All variables were calculated for conventional (longitudinal, circumferential, and radial) and principal directions (minimum and maximum) of deformation, with regional wall thickness calculated in the radial direction (1, 2, 9). Data were reported for subendocardial, subepicardial, and transmural layers, as appropriate.

**Figure 2:**
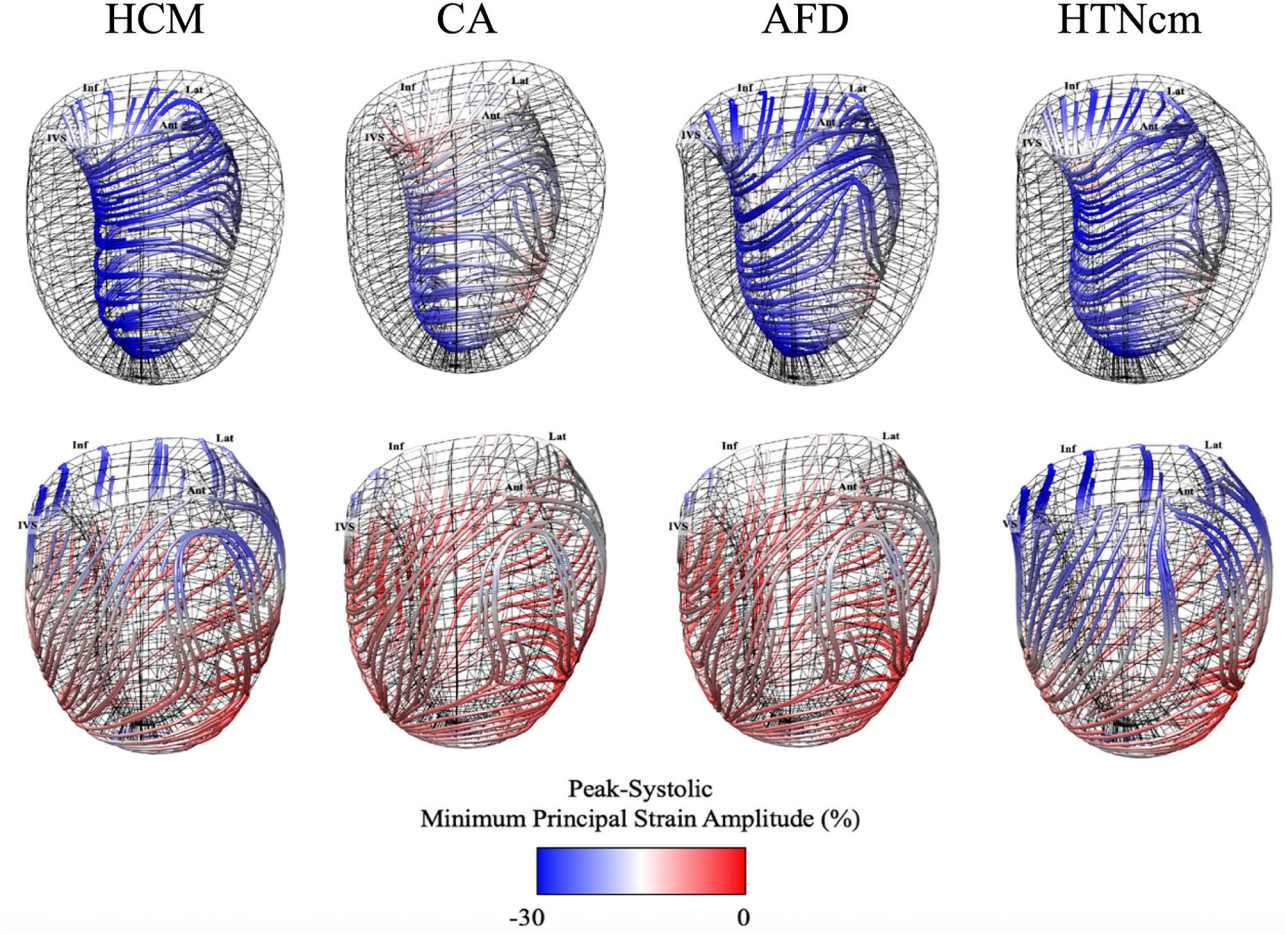
Average minimum principal strain and direction average atlases for patients with hypertrophic cardiomyopathy (HCM: *N* = 85), cardiac amyloidosis (CA: *N* = 18), Anderson–Fabry Disease (AFD: *N* = 30), and Hypertensive cardiomyopathy (HTNcm: *N* = 30) (9).

Figure 2 provides atlas-based representations of 3D-MDA features that were used to predict disease etiology, simultaneously presenting architectural and deformation-based features used in the classifier. Atlases have been rendered at peak-systole frame with color-coding of peak strain amplitude (minimum principal strain) along directional lines of deformation. The latter aims to provide a visual representation of the intrinsic (local) direction of dominant tissue deformation as described by minimum principal strain (1), this being a resulting effect of myofibril engagement between the endocardial and epicardial layers (9).

### Regional-Pattern-Based Classifier

A feed-forward neural network model was used as classifier. We aimed to capture composite features indicative of HCM phenotype vs. any other disease phenocopy state (CA, AFD or HTNcm) using 3D-MDA data. Our goal was a ML-based model that could use 917 segmental architectural and deformation-based features. These features were provided as AHA segmental, layer-specific (i.e., subepicardial, subendocardial, and transmural) systolic amplitude, time to systolic peak, peak-systolic rate, peak-diastolic strain rate, and wall thickness. Each AHA segmental feature was obtained by averaging values across all nodes within the corresponding AHA segment. To develop the ML-based model we used a neural network taking all 917 features as inputs. Two fully-connected hidden layers (30 and 5 neurons, respectively, each using a hyperbolic tangent sigmoid transformation) were used. A final read-out single-perceptron layer, followed by a log-sigmoid activation function, provided a real-valued output between zero and one to predict the presence of HCM (assigned to HCM when >0.5).

The choice of architecture (i.e., number of neurons in each layer, and number of hidden layers) was based on using, for each layer, the square root of the number of features from the previous layer, in order to establish a proof of concept model. Following this criterion to increasingly reduce the number of features throughout the network, only two hidden layers were deemed necessary. Our learner used a cross-entropy loss function as a reference point for the backward pass, and trained for 100 epochs.

The full pipeline from contouring to meshing, tracking, strain analysis, and neural network building can be replicated following the previously described 3D-MDA methodology (1), and building a fully-connected network according to the above specified hyperparameters for size of the layers, network depth, and used activation functions. Image acquisition parameters are provided within the “MRI Image acquisition” section.

While preferable to use one set of instances for training and a holdout subset to estimate the accuracy of the learned model, given our small sample size, we estimated diagnostic performance using a 5-fold cross-validation procedure (19). This involved using 4/5-ths of the patients to develop a model whose performance was evaluated on the remaining 1/5-th of patients, which were not previously seen by the training process. This procedure was repeated five times, rotating the 4/5-th training portion and 1/5-th testing portion. It is worth noting that, on each fold, feature selection and parameter estimation (training) were performed from scratch on the first 4/5-ths, thus relegating the last 1/5-th to an external holdout at each iteration, and reducing the chance of overfitting.

Performance was evaluated as area under the curve (AUC) of the receiver operating characteristic (ROC) curve as well as respective measures of sensitivity, specificity, positive and negative predictive values, and accuracy. These values were reported as mean with standard deviation range derived from the 5-fold cross-validation procedure (19). Training and validation procedures were implemented *via* Matlab Statistics and Machine Learning Toolbox (R2019b, The Mathworks, Inc., Natick, MA, USA).

### Statistical Analysis

Categorical variables were presented as counts with percentages, while continuous variables were expressed as mean with standard deviation. Categorical variables were compared using the Fisher's exact test, and comparisons for continuous data were performed with the 2-sample *t*-test or Mann-Whitney U test, where appropriate. A two-sided *p*-value of <0.05 was considered statistically significant. Multiple comparisons between patients stratified by phenocopy disease state were performed by ANOVA. All statistical analysis was performed using the Matlab Statistics and Machine Learning Toolbox (R2019b, The Mathworks, Inc., Natick, MA, USA).

## Results

### Study Population

A total of 163 patients with LVH of known etiology were studied (85 HCM, 30 AFD, 30 HTNcm, 18 CA). Patient characteristics are provided in Table 1. The mean age was 52.7 ± 14.7 years, with 67 females (41%). No significant differences in baseline clinical characteristics were observed between HCM and non-HCM patients, with the exception of age and BSA. However, neither of these two variables provided significant predictive value for the classification of HCM (AUC = 0.63 for each). The following count was found for the HCM sub-phenotypes: (i) isolated basal-septal: 57 (67%), (ii) reverse septal curvature: 12 (14%), (iii) apical: 11 (13%), and (iv) other sub-phenotypes: 5 (6%).

**Table 1:** Clinical characteristics of all patients with left ventricular hypertrophy (LVH), patients with LVH and confirmed etiology of hypertrophic cardiomyopathy (LVH+ HCM+) and patients with LVH and a mimicking cardiomyopathy state (LVH+ HCM–).

		**LVH+HCM+**	**LVH+HCM–**					
	**All patients**	**HCM**	**All LVH+HCM–**	**CA**	**AFD**	**HTNcm**		
**Parameter**	**(*N* = 163)**	**(*N* = 85)**	**(*N* = 78)**	**(*N* = 18)**	**(*N* = 30)**	**(*N* = 30)**	***p*-Value**	**AUC (95% CI)**
**Clinical characteristics**								
Age, years	52.7 ± 14.7	50.0 ± 11.3	55.7 ± 17.4^**^	66.8 ± 16.4^****^	45.1 ± 16.2	59.6 ± 13.1^***^	<**0.0001**	0.63 (0.59 –0.67)
Female, *n* (%)	67 (41)	37 (47)	30 (35)	4 (22)	19 (63)^**^	14 (47)	–	–
BSA (m^2^)	2.0 ± 0.3	2.1 ± 0.3	2.0 ± 0.3^**^	2.0 ± 0.3	1.9 ± 0.2^***^	2.0 ± 0.3	<**0.005**	0.63 (0.53–0.74)
Hypertension, *n* (%)	67 (41)	36 (46)	31 (36)	5 (29)	3 (12)^*^	28 (93)	–	–
Hyperlipidemia, *n* (%)	25 (15)	14 (18)	11 (13)	2 (12)	2 (8)	10 (33)	–	–
Diabetes, *n* (%)	19 (12)	9 (12)	10 (12)	1 (6)	2 (7)	6 (20)	–	–
Smoking, *n* (%)	62 (38)	27 (35)	35 (41)	6 (33)	16 (53)	5 (17)^*^	–	–
**ECG parameters**								
LBBB, *n* (%)	11 (7)	4 (5)	7 (8)	2 (11)	0 (0)	2 (7)	–	–
QRS duration (ms)	99.3 ± 17.4	98.3 ± 16.6	100.9 ± 18.7	102.4 ± 24.3	105.1 ± 15.1	96.2 ± 14.2	–	–
**NYHA functional class**								
Class I, *n* (%)	47 (29)	25 (32)	22 (26)	8 (44)	7 (23)	10 (33)	–	–
Class II, *n* (%)	26 (16)	13 (17)	13 (15)	4 (22)	5 (17)	4 (13)	–	–
Class III, *n* (%)	18 (11)	8 (10)	10 (12)	3 (17)	0 (0)	5 (17)	–	–
Class IV, *n* (%)	3 (2)	2 (3)	1 (1)	0 (0)	1 (3)	1 (3)	–	–
**Medications**								
ACE inhibitors or ARB, *n* (%)	52 (32)	31 (40)	21 (25)^*^	7 (39)	7 (23)	17 (57)^**^	–	0.56 (0.45–0.67)
Amiodarone, *n* (%)	4 (2)	2 (3)	2 (2)	1 (6)	1 (3)	0 (0)	–	–
Beta-blocker, *n* (%)	71 (44)	26 (33)	45 (53)^*^	3 (17)^**^	6 (20)^**^	17 (57)	–	0.60 (0.53–0.67)
Digoxin, *n* (%)	1 (1)	1 (1)	0 (0)	0 (0)	0 (0)	1 (3)	–	
Diuretic, *n* (%)	39 (24)	25 (32)	14 (16)^*^	11 (61)^***^	4 (13)	10 (33)	–	0.58 (0.55–0.61)
Statin, *n* (%)	51 (31)	23 (29)	28 (33)	5 (28)	4 (13)	14 (47)	–	–

*AFD, Anderson–Fabry disease; BSA, body surface area; ECG, electrocardiogram; CA, cardiac amyloidosis; HCM, hypertrophic cardiomyopathy; HTNcm, hypertensive hypertrophy; LVH, left-ventricular hypertrophy; LBBB, left bundle branch block; NYHA, New York Heart Association*.

Vs. HCM:

* *<0.05*.

** *<0.01*.

*** *<0.001*.

**** *<0.0001*.

*Bold values are used for specific headers or p-Values lower than 0.05 (i.e. statistical significance)*.

### Disease Classification Using Conventional Volumetric Cine Imaging Markers

Of all conventionally reported CMR characteristics (Table 2), significant differences between HCM and non-HCM etiology were identified for LVEDVI (81.0 ± 15.1 mL/m^2^ vs. 75.5 ± 20.4 mL/m^2^, respectively— *p* < 0.01), LVEF (70.0 ± 7.5% vs. 65.4 ± 10.7%, respectively— *p* < 0.05), LVMI (84.2 ± 26.3 g/m^2^ vs. 75.9 ± 21.8 g/m^2^, respectively— *p* < 0.05), RVESVI (28.1 ± 8.8 mL/m^2^ vs. 35.6 ± 17.4 mL/m^2^, respectively— *p* < 0.01), RVEF (62.8 ± 7.5 ± vs. 57.7% ± 9.7%, respectively— *p* < 0.001), and LAEDVI (103.7 ± 38.5 mL/m^2^ vs. 78.0 ± 26.3 mL/m^2^, respectively —*p* < 0.0001). The highest AUC achieved of all such variables was for LAEDVI, reaching 0.70 (0.54— 0.90).

**Table 2:** CMR-based volumetric measurements of all patients with left ventricular hypertrophy (LVH), patients with LVH and confirmed etiology of hypertrophic cardiomyopathy (LVH+ HCM+) and patients with LVH and a mimicking cardiomyopathy state (LVH+ HCM–).

		**LVH+ HCM+**	**LVH+HCM–**					
	**All patients**	**HCM**	**All LVH+HCM–**	**CA**	**AFD**	**HTNcm**		
**Parameter**	**(*N* = 163)**	**(*N* = 85)**	**(*N* = 78)**	**(*N* = 18)**	**(*N* = 30)**	**(*N* = 30)**	***p*-Value**	**AUC (95% CI)**
LVEDVI (mL/m^2^)	78.4 ± 18.0	81.0 ± 15.1	75.5 ± 20.4^**^	80.8 ± 29.6	75.0 ± 15.3	72.9 ± 18.3^*^	0.1	0.65 (0.51–0.80)
LVESVI (mL/m^2^)	25.7 ± 12.2	24.6 ± 8.4	27.0 ± 15.3	37.0 ± 25.3^*^	25.1 ± 8.4	22.9 ± 9.5	0.1	–
LVEF (%)	67.8 ± 9.4	70.0 ± 7.5	65.4 ± 10.7^*^	56.4 ± 15.6^***^	66.8 ± 6.5	69.3 ± 7.4	**0.001**	0.60 (0.50–0.70)
LVMI (g/m^2^)	80.2 ± 24.6	84.2 ± 26.3	75.9 ± 21.8^*^	84.2 ± 28.3	65.4 ± 17.3^***^	81.5 ± 17.6	**<0.005**	0.60 (0.49–0.70)
RVEDVI (mL/m^2^)	78.5 ± 20.7	75.1 ± 15.7	82.2 ± 24.6	91.4 ± 32.5^**^	79.8 ± 19.8	78.9 ± 22.9	0.3	–
RVESVI (mL/m^2^)	31.7 ± 14.1	28.1 ± 8.8	35.6 ± 17.4^**^	46.4 ± 25.7^***^	31.9 ± 12.1	32.9 ± 13.1	**0.005**	0.62 (0.49–0.70)
RVEF (%)	60.4 ± 9.0	62.8 ± 7.5	57.7 ± 9.7^***^	51.1 ± 11.5^****^	60.8 ± 8.0	58.7 ± 8.3^*^	**<0.0001**	0.68 (0.54–0.80)
LAEDVI (mL/m^2^)	91.5 ± 35.6	103.7 ± 38.5	78.0 ± 26.3^****^	89.4 ± 27.6	66.5 ± 20.4^****^	83.0 ± 27.2^**^	**<0.0001**	0.70 (0.54–0.90)

*AFD, Anderson-Fabry Disease; CA, cardiac amyloidosis; HCM, hypertrophic cardiomyopathy; HTNcm, hypertensive hypertrophy; LAEDVI, left-atrial end-diastolic volume; LVEDVI, left-ventricular end-diastolic volume index; LVEF, left-ventricular ejection fraction; LVH, left-ventricular hypertrophy; LVMI, left-ventricular mass index; LVESVI; left-ventricular end-systolic volume index; RVEDVI, right-ventricular end-diastolic volume index; RVEF, right-ventricular ejection fraction; RVESVI, right-ventricular end-systolic volume index*.

Vs. HCM:

* *<0.05*.

** *<0.01*.

*** *<0.001*.

**** *<0.0001*.

*Bold values are used for specific headers or p-Values lower than 0.05 (i.e. statistical significance)*.

### Disease Classification Using Global Strain Amplitude and Time to Peak Systolic Strain

To establish a baseline, we studied the capacity of conventional strain-based measures to classify disease. Global peak-systolic strain amplitude values of the study population are presented in Table 3. Of conventional, axis-dependent measures of strain, global circumferential strain amplitude measured at the subendocardial layer provided the highest AUC for the classification of HCM vs. non-HCM etiology with an AUC of 0.62 (0.54—0.71). Global minimum principal strain measured at the subendocardial layer showed similar performance with an AUC of 0.64 (0.52—0.76), whereas transmurally the AUC was documented at 0.60 (0.49—0.71) in the radial direction.

**Table 3:** Global peak systolic strain amplitude and end-diastolic wall thickness of all patients with left ventricular hypertrophy (LVH), patients with LVH and confirmed etiology of hypertrophic cardiomyopathy (LVH+HCM+) and patients with LVH and a mimicking cardiomyopathy state (LVH+ HCM–).

		**LVH+HCM+**	**LVH+HCM–**					
	**All patients**	**HCM**	**All LVH+HCM–**	**CA**	**AFD**	**HTNcm**		
**Global parameter**	**(*N* = 163)**	**(*N* = 85)**	**(*N* = 78)**	**(*N* = 18)**	**(*N* = 30)**	**(*N* = 30)**	***p*-Value**	**AUC (95% CI)**
**Circumferential**								
Subendocardium (%)	−17.1 ± 3.9	−17.8 ± 3.7	−16.3 ± 4.0^*^	−14.2 ± 5.9^***^	−16.5 ± 2.6	−17.2 ± 3.5	**<0.001**	0.62 (0.54–0.71)
Subepicardium (%)	−6.8 ± 1.9	−6.9 ± 1.9	−6.8 ± 2.0	−6.0 ± 2.2	−7.1 ± 1.9	−6.9 ± 1.9	0.29	–
Transmural (%)	−11.2 ± 2.7	11.5 ± 2.6	−11.0 ± 2.7	−9.5 ± 3.7^**^	−11.4 ± 2.0	−11.5 ± 2.5	**<0.05**	–
**Longitudinal**								
Subendocardium (%)	−15.0 ± 3.8	−15.2 ± 3.9	−14.7 ± 3.7	−12.6 ± 5.1^*^	−15.3 ± 2.0	−15.3 ± 3.8	0.06	–
Subepicardium (%)	−6.6 ± 2.4	−6.5 ± 2.3	−6.7 ± 2.4	−5.7 ± 2.3	−7.3 ± 2.6	−6.6 ± 2.1	0.1	–
Transmural (%)	−10.3 ± 2.7	−10.4 ± 2.8	−10.2 ± 2.7	−8.6 ± 3.3^*^	−11.0 ± 1.7	−10.4 ± 2.7	**<0.05**	–
Global longitudinal shortening (%)	−13.0 ± 4.2	−12.7 ± 4.2	−13.4 ± 4.2	−10.5 ± 5.0^*^	−14.9 ± 2.8^*^	−13.6 ± 4.0	**<0.005**	–
**Radial**								
Transmural (%)	40.6 ± 19.6	37.4 ± 18.0	44.0 ± 20.9^*^	29.5 ± 19.1	49.8 ± 17.1^**^	46.8 ± 21.8^*^	**<0.0005**	0.60 (0.49–0.71)
**Minimum principal**								
Subendocardium (%)	−26.8 ± 4.7	−27.8 ± 4.2	−25.7 ± 5.1^**^	−22.2 ± 7.2^****^	−27.0 ± 3.0	−26.4 ± 4.3	**0.0001**	0.64 (0.52–0.76)
Subepicardium (%)	−18.5 ± 3.5	−18.6 ± 3.1	−18.3 ± 3.9	−14.7 ± 4.6^****^	−20.3 ± 2.6^**^	−18.5 ± 3.0	**0.0001**	–
Transmural (%)	−22.8 ± 4.1	−23.4 ± 3.6	−22.2 ± 4.6	−18.5 ± 6.2^****^	−24.0 ± 2.7	−22.6 ± 3.8	**<0.0001**	–
**Maximum transmural**								
Transmural (%)	64.7 ± 24.5	63.2 ± 21.5	66.3 ± 27.6	46.1 ± 26.2^**^	77.0 ± 21.6^**^	67.8 ± 27.8	**<0.0005**	–
Thickness (mm)	10.3 ± 2.5	11.3 ± 2.3	9.1 ± 2.1^****^	10.7 ± 2.7	8.1 ± 1.9^****^	9.3 ± 1.2^****^	**<0.0001**	0.75 (0.71–0.80)

*AFD, Anderson-Fabry Disease; CA, cardiac amyloidosis; CI, confidence interval; HCM, hypertrophic cardiomyopathy; HTNcm, hypertensive hypertrophy; LVH, left-ventricular hypertrophy*.

Vs. HCM:

* *<0.05*.

** *<0.01*.

*** *<0.001*.

**** *<0.0001*.

*Bold values are used for specific headers or p-Values lower than 0.05 (i.e. statistical significance)*.

Time to peak-systolic strain amplitude data (indexed by duration of the cardiac cycle) is reported in Table 4. In the circumferential direction, HCM and non-HCM etiology were significantly different in strain timing, both when measured subendocardially (41.4 ± 5.1% vs. 43.2 ± 5.9%, respectively— *p* < 0.05) and transmurally (40.6 ± 5.0 ± 42.7 ± 5.6, respectively— *p* < 0.01). The latter achieved the highest timing-related AUC of 0.62 (0.53—0.72).

**Table 4:** Global time to peak systolic strain (% of the cardiac cycle length) of all patients with left ventricular hypertrophy (LVH), patients with LVH and confirmed etiology of hypertrophic cardiomyopathy (LVH+HCM+) and patients with LVH and a mimicking cardiomyopathy state (LVH+HCM–).

		**LVH+HCM+**	**LVH+HCM**–					
	**All patients**	**HCM**	**All LVH+HCM–**	**CA**	**AFD**	**HTNcm**		
**Global parameter**	**(*N* = 163)**	**(*N* = 85)**	**(*N* = 78)**	**(*N* = 18)**	**(*N* = 30)**	**(*N* = 30)**	***p*-Value**	**AUC (95% CI)**
**Circumferential**								
Subendocardium (%)	42.3 ± 5.6	41.4 ± 5.1	43.2 ± 5.9^*^	41.4 ± 6.5	42.4 ± 4.8	45.2 ± 6.1^**^	**<0.05**	0.59 (0.50–0.68)
Subepicardium (%)	40.2 ± 5.6	39.5 ± 5.2	40.9 ± 5.9	39.4 ± 6.1	40.1 ± 5.1	42.6 ± 6.4^**^	0.1	–
Transmural (%)	41.6 ± 5.4	40.6 ± 5.0	42.7 ± 5.6^**^	41.2 ± 5.8	41.6 ± 4.6	44.7 ± 6.1^***^	**<0.01**	0.62 (0.53–0.72)
**Longitudinal**								
Subendocardium (%)	42.6 ± 5.1	42.5 ± 5.2	42.8 ± 5.1	42.4 ± 7.2	42.0 ± 4.5	43.8 ± 4.2	0.5	–
Subepicardium (%)	41.4 ± 5.4	41.3 ± 5.6	41.6 ± 5.2	40.3 ± 6.6	41.4 ± 4.6	42.7 ± 4.9	0.5	–
Transmural (%)	42.2 ± 5.1	42.0 ± 5.3	42.5 ± 5.0	42.0 ± 6.0	41.8 ± 4.4	43.5 ± 4.8	0.6	–
**Radial**								
Transmural (%)	43.6 ± 5.6	43.5 ± 5.3	43.7 ± 6.0	43.0 ± 8.1	42.1 ± 4.6	45.7 ± 5.5	0.1	–
**Minimum principal**								
Subendocardium (%)	41.4 ± 4.8	41.7 ± 4.6	41.1 ± 4.9	39.9 ± 5.7	40.7 ± 4.2	42.3 ± 5.1	0.3	–
Subepicardium (%)	41.0 ± 4.8	41.4 ± 5.0	40.6 ± 4.5	39.4 ± 6.8	40.6 ± 3.7	41.4 ± 3.5	0.4	–
Transmural (%)	41.1 ± 4.7	41.5 ± 4.7	40.6 ± 4.6	39.4 ± 6.1	40.5 ± 4.0	41.3 ± 4.2	0.4	–
**Maximum transmural**								
Transmural (%)	43.3 ± 5.0	43.1 ± 4.9	43.6 ± 5.2	43.2 ± 6.8	42.4 ± 4.3	45.0 ± 4.8^*^	0.1	–

*AFD, Anderson–Fabry disease; CA, cardiac amyloidosis; CI, confidence Interval; HCM, hypertrophic cardiomyopathy; HTNcm, hypertensive hypertrophy; LVH, left-ventricular hypertrophy*.

Vs. HCM:

* *<0.05*.

** *<0.01*.

*** *<0.001*.

**** <0.0001.

*Bold values are used for specific headers or p-Values lower than 0.05 (i.e. statistical significance)*.

### Disease Classification Using Regional Wall Thickness

The sole use of architectural features to classify disease was explored, this considering segmental end-diastolic mean wall thickness derived from each 3D-MDA mesh-based model. This led to an AUC of 0.75 (0.71–0.80) for the discrimination of HCM vs. non-HCM etiology (Table 3).

### Neural-Net-Based Disease Classification Using 3D-MDA-Derived Data

**Figure 3:**
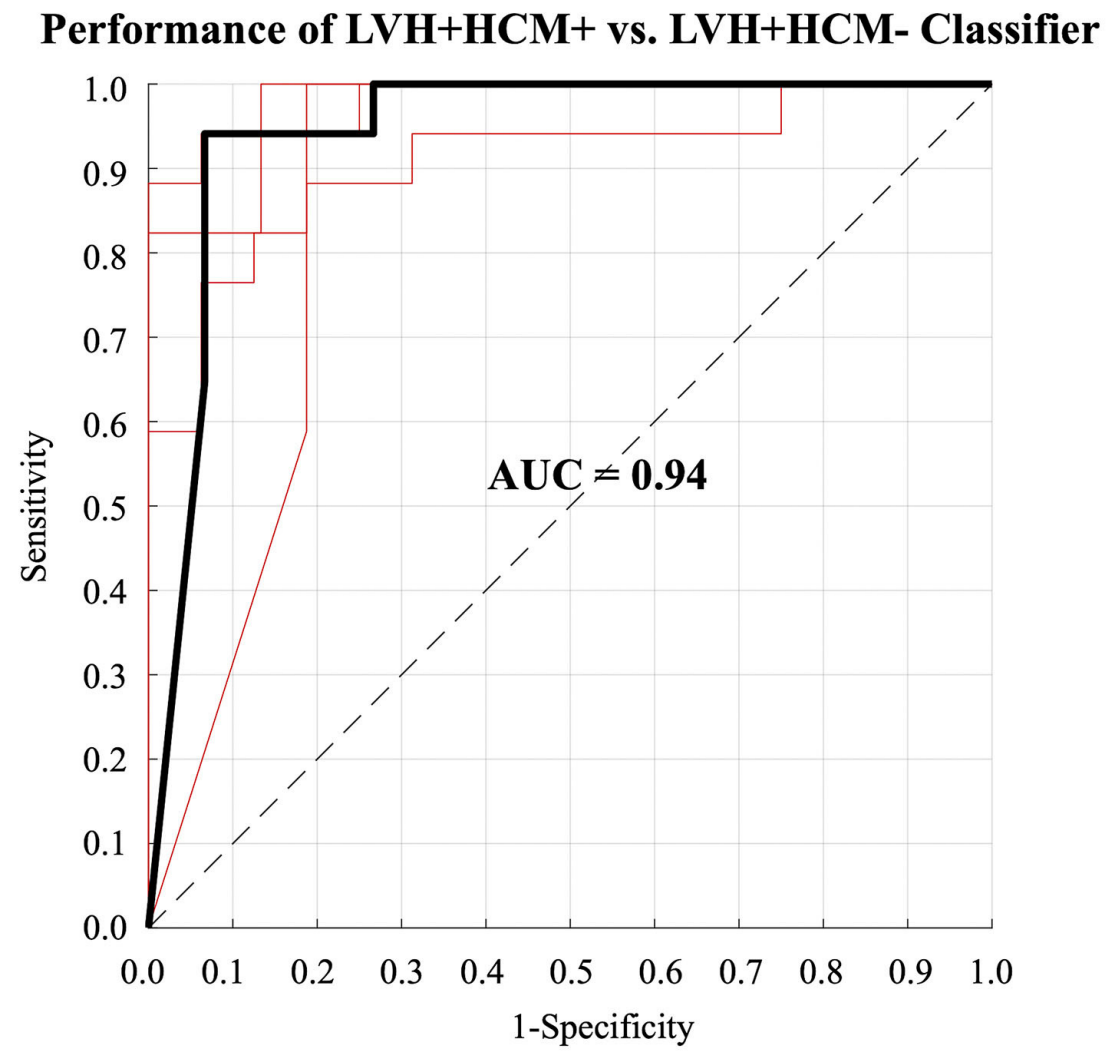
Receiver operating characteristic (ROC) curves for the classification of patients with left ventricular hypertrophy (LVH) due to hypertrophic cardiomyopathy (LVH+HCM+) obtained during the 5-fold cross validation. The heavy black line indicates the median ROC curve.

Finally, neural network-based classification performance of 3D-MDA data was assessed using all available architectural and deformation features, inclusive of regional measures of wall thickness, strain amplitude and time to peak-systolic strain. A neural network-based model, developed from all AHA segmental architectural and deformation-based features, demonstrated diagnostic performance with a mean AUC (ROC) of 0.94 (0.89–0.99) for the classification of HCM etiology, as shown in Figure 3. Mean sensitivity and specificity for HCM etiology was 0.92 (0.85–0.98) and 0.90 (0.83–0.97), respectively, with the mean accuracy being 0.91 (0.87–0.95). Mean positive and negative predictive values were 0.91 (0.85–0.97) and 0.91 (0.85−0.98), respectively.

## Discussion

This study demonstrates capacity for ML-based methodologies to execute disease classification tasks, when provided a highly standardized set of myocardial architectural and deformation features, in this case through 3D-MDA of routine non-contrast cine CMR.

To our knowledge, this is the first study in the literature demonstrating feasibility of neural networks to classify the diagnosis of a hypertrophic cardiomyopathy state using 3D measures of myocardial architecture and deformation. Our neural network was trained using over 900 phenotypic features that were generated by 3D-MDA, this providing intrinsic temporo-spatial data registration across individual subjects. Using this structured data, we were able to deliver good performance using a two-layer neural network, achieving high discriminatory performance (AUC 0.94) for classifying HCM from its mimic cardiomyopathy states.

A single prior study has evaluated ML-based classification of HCM using 2D-based measures of deformation, these derived from speckle-tracking echocardiography (5). In this study, reported by Narula et al. (5), a classifier was trained to differentiate HCM from the presumptive diagnosis of athletic heart, achieving an AUC of 0.795. In contrast to this study, ours examined 3D features of architecture and deformation among clinically confirmed pathologic causes of hypertrophy. Furthermore, we compared model performance to the performance based on other clinically available markers, inclusive of clinical referral data, non-strain based volumetric CMR data, global strain-based markers, and 3D-MDA based measures modeled without ML techniques. We observed modest performance for conventional global strain-based measures to differentiate HCM from common phenocopy states. This finding is similar to a prior report by Neisius et al. (20), who attempted to distinguish HCM from HTNcm subjects and identified similar performance. By contrast, we demonstrated substantial gains in diagnostic accuracy for ML-based algorithms trained from 3D-MDA.

Dawes et al. (21) has previously described disease classification from 3D modeled descriptors of deformation for the right ventricle (RV). In this study, they elegantly demonstrated an atlas-based approach to perform RV segmentation from SAX cine images followed by modeling of chamber deformation (21). This allowed for construction of a dynamic 3D mesh model to derive RV deformation features in conventional axis-dependent directions of deformation. Following principal component analysis for feature reduction, this framing a regression-based approach, they trained models to identify patients at elevated risk of clinical outcomes, demonstrating superior performance to conventional CMR-based markers. This study highlighted a potential role for 3D myocardial deformation analyses to provide relevant information for ML-based tool development. The same research group reinforced this concept in predicting survival among a cohort of 302 patients with pulmonary hypertension (22).

While deformation is considered central to the diagnosis of cardiomyopathies, Swift et al. (23) explored an alternative approach to perform feature extraction directly from static (single phase) 2D cine CMR images. In this work a tensor-based machine-learned model was developed to evaluate pixel-based features from SAX and 4-chamber images, demonstrating the ability to classify presence vs. absence of pulmonary hypertension with good accuracy. However, this model did not perform significantly better than conventional CMR-based markers. How such methods will facilitate the differentiation of similar diseases remains uncertain at this time.

Our observation that regional deformation features provide incremental value to global architectural and global functional features in classifying disease etiology is consistent with emerging evidence that such measures are intimately coupled to underlying tissue characteristics (24). Strain-based markers have shown strong regional correlation with underlying markers of fibrosis (i.e., interstitial expansion) among patients with HCM (2, 25–28) and CA (13, 29), as well as in non-hypertrophic states such as ischemic (30–32) and dilated cardiomyopathy (33). Accordingly, capacity exists for regionally encoded markers of deformation to provide unique insights into underlying myocardial tissue health, and in-turn provide relevant information for the classification of disease etiology.

The use of routine strain-based markers to assist in the discrimination of hypertrophic disease states has been explored using both echocardiography (34–36), and CMR-based (37) techniques. Williams et al. demonstrated that 2D strain analysis of cine CMR data shows greater relative preservation of apical global longitudinal strain in CA vs. HCM and AFD (37), and this pattern of apical sparing has similarly been described across echocardiography-based studies (38–40). Reduction in global longitudinal strain have also been shown in biopsy proven HCM relative to hypertensive hypertrophy (36). While these studies have aimed to deliver simple and practical “rules of thumb” for identifying patients at greater likelihood of a specific disease, incremental value is inherently provided through ML-based modeling of raw deformation data, allowing for non-linear relationships to be identified across a larger number of features.

### Study Limitations

Our study has several limitations. Our training cohort was modest in size and based on single-center data. As a consequence, we decided to contain model complexity by constructing it with only fully-connected hidden layers. Accordingly, our model may benefit from training dataset expansion and requires external validation in a multi-center clinical setting. Our evaluation was based on 5-fold cross-validation, as a separate hold-out validation cohort was not available. To address this, for each of the 5 cross-validations, training was restricted to the first 4/5-ths of the population followed by external validation on a 1/5-th hold-out population, this being an effective strategy to reduce the chance of overfitting. However, generalizability of our model to other patient populations (i.e., from other centers) has not been assessed. We chose to describe each patient using only segmentally-coded data from 3D-MDA in our neural network, despite having access to features derived for each hexahedral element of the mesh model. This decision was based on the size of our available population and practical application of the AHA segmental model as a feature reduction strategy. Consistent with clinical practice, we did not mandate genotype positivity for establishing a clinical diagnosis of HCM, rather also accepted phenotype positive patients if they had a first-degree family member with positive genotype or had a typical LGE-based pattern of fibrosis with no alternative explanatory disease. While potentially excluding mild HCM phenotypes without genotypic or LGE criteria, we aimed to maximize robustness of diagnostic classification for all LVH etiologies. The size of our neural-network was small and we used a simple fully-connected architecture to drastically reduce the number of features from each layer to the next one. This approach was taken to address the modest size of the available population and limit the number of parameters to be trained within the network. With exposure to larger populations, deeper networks with less aggressive feature reduction between layers are possibly applicable, and this is a priority for future studies. However, despite such limitations, strong discriminatory performance was achieved to distinguish HCM from non-HCM disease states in this study. We acknowledge that a significant difference in age and sex was observed between the respective disease cohorts, and that the influence of this on biomechanical disease profiles remains unknown. How training cohort demographics influence generalizability of ML-based diagnostic tools is critically important to be address in future studies. Finally, in anticipation of reductions in image quality, we *a-priori* excluded patients in atrial fibrillation. Accordingly, we cannot confirm performance of the described ML-based model for patients not in sinus rhythm.

### Conclusions

We have demonstrated feasibility and acceptable performance of a neural network-based approach for the automated discrimination of HCM vs. its known phenocopy states. This unique approach, leveraging standardized phenotypic data from 3D-MDA, offers expanded potential for ML-assisted diagnostics and justifies broader investigation for other disease phenotypes.

## Data Availability Statement

The datasets presented in this article are not readily available because the data underlying this article will be shared on reasonable request. Requests to access the datasets should be directed to James A. White, jawhit@ucalgary.ca.

## Ethics Statement

The studies involving human participants were reviewed and approved by Conjoint Health Research Ethics Board at the University of Calgary. Written informed consent to participate in this study was provided by the participants' legal guardian/next of kin.

## Author Contributions

AS and JW contributed to conception and design of the study. AS contributed software and model development. SD and RG assisted with model development. YA, MS, and CW made measurements. PF assisted with image acquisition. RS contributed with patient recruitment. JF and JW acquired ethics approval. AS performed the statistical analysis, model assessment, and drafted the manuscript. AF, BH, NM, AH, CL, SD, AK, RG, and JW revised the manuscript critically for important intellectual content. All authors provided approval for publication of the manuscript.

## Conflict of Interest

JF, AH, and JW are shareholders of Cohesic, Inc. AK is a member of the Canadian Fabry Disease Initiative (CFDI). AK and the CFDI have received sponsorship from any combination of Sanofi Genzyme, Takeda, and Amicus as research grants. The data contributed to this study were not collected as part of a clinical trial, under any form of sponsorship nor part of government or commercially funded enterprise, and utilized for academic purposes only. The remaining authors declare that the research was conducted in the absence of any commercial or financial relationships that could be construed as a potential conflict of interest.
